# Donor-Derived CD123-Targeted CAR T Cell Serves as a RIC Regimen for Haploidentical Transplantation in a Patient With *FUS-ERG*+ AML

**DOI:** 10.3389/fonc.2019.01358

**Published:** 2019-12-03

**Authors:** Sun Yao, Chen Jianlin, Liu Yarong, Li Botao, Wang Qinghan, Fang Hongliang, Zhang Lu, Ning Hongmei, Wang Pin, Chen Hu, Hu Liangding, Zhang Bin

**Affiliations:** ^1^Department of Hematopoietic Stem Cell Transplantation, The Fifth Medical Center of Chinese PLA General Hospital (Former 307th Hospital of PLA), The Research Institute of Hematopoietic Stem Cell of the People's Liberation Army, Beijing, China; ^2^R&D Department, HRAIN Biotechnology Co., Ltd., Shanghai, China; ^3^Department of Biomedical Engineering, University of Southern California, Los Angeles, CA, United States; ^4^Department of Chemical Engineering and Materials Science, University of Southern California, Los Angeles, CA, United States; ^5^Department of Pharmaceutical Sciences and Pharmacology, University of Southern California, Los Angeles, CA, United States; ^6^Beijing Key Laboratory of Hematopoietic Stem Cell Therapy and Transformation Research, Department of Hematopoietic Stem Cell Transplantation, The Cell and Gene Therapy Center, The Fifth Medical Center of Chinese PLA General Hospital (Former 307th Hospital of PLA), The Research Institute of Hematopoietic Stem Cell of the People's Liberation Army, Beijing, China

**Keywords:** chimeric antigen receptor, CD123, allogeneic hematopoietic stem cell transplantation, acute myeloid leukemia, cytokine release syndrome, graft-vs-host disease, *FUS-ERG*

## Abstract

**Background:** Allogeneic hematopoietic stem cell transplantation (allo-HSCT) following chemotherapy is part of standard treatment protocol for patients with acute myeloid leukemia (AML). *FUS-ERG*+ AML is rare but has an extremely poor prognosis even with allo-HSCT in remission, possibly due to its a leukemia stem cell (LSC)-driven disease resulting in chemotherapy resistance and a novel therapy is urgently required. It has been reported that *FUS-ERG*-positive AML expresses CD123, a marker of LSC, in some cases. CD123-targeted CAR T cell (CART123) is promising immunotherapy, but how to improve the complete remission (CR) rate and rescue potential hematopoietic toxicity still need to explore.

**Case Presentation:** We used donor-derived CART123 as part of conditioning regimen for haploidentical HSCT (haplo-HSCT) in a patient with *FUS-ERG*+ AML who relapsed after allogeneic transplantation within 3 months, resists to multi-agent chemotherapy and donor lymphocyte infusion (DLI) and remained non-remission, aiming to reduce these chemotherapy-resistant blasts and rescue potential hematopoietic toxicity. The blasts in BM were reduced within 2 weeks and coincided with CAR copies expansion after CART123 infusion. The patient achieved full donor chimerism, CR with incomplete blood count recovery, and myeloid implantation.

**Conclusion:** Our results hints that CART123 reduces the chemotherapy-resistant AML blasts for *FUS-ERG*+ AML without affecting the full donor chimerism and myeloid implantation.

## Introduction

Allogeneic hematopoietic stem cell transplantation (allo-HSCT) is part of standard treatment protocol for patients with high-risk acute myeloid leukemia (AML). In some cases, however, even allo-HSCT in remission still could not overcome the poor prognosis, *FUS-ERG*+ AML patients are one of them (1–3). *FUS-ERG* fusion gene is formed by the translocation t(16;21) (p11;q22), which is a rare reciprocal chromosomal change. This translocation has been most frequently reported in AML, with an incidence of 1% (4). To date, more than 100 patients with *FUS-ERG*+ AML have been described in patients from 1 to about 60 years of age, of which most are children (1–3, 5). In childhood, *FUS-ERG*+ AML patients who achieved MRD-negative showed no significant difference in event-free survival (EFS) compared to that of MRD-positive, possibly due to its a leukemia stem cell (LSC)-driven disease and cannot be successfully eradicated with current treatment protocol (2). Moreover, 4 years EFS of *FUS-ERG*+ AML is 7%, while the high-risk group is 45%, indicating an inferior prognosis of *FUS-ERG*+ AML (2). Furthermore, the *FUS-ERG*+ AML patients with leukemia burden before transplantation even had a poorer prognosis than those with complete remission (CR) (1). Thus, these patients urgently require novel forms of therapy.

Until now, CD123 positive in *FUS-ERG*+ AML patients was reported in several studies (1, 6–8). CD123 is expressed in 40–93% of patients with AML and is one of the significant markers of LSC when expressed at meager amounts or not found in healthy CD34+ hematopoietic cells (9, 10). Given increased research on LSC in the past two decades, researchers found that LSC is quiescent for a long time and the possible origin of leukemic blasts, which represent critical factors for chemotherapy resistance (11). This finding makes CD123 one of the most promising targets for AML treatment. Also, CD123 is generally expressed in some myeloid progenitor cells, monocytes, plasmacytoid dendritic cells (pDC), basophils, and endothelial cells (12, 13).

Chimeric antigen receptor (CAR) T cell targeting CD19 have demonstrated remarkable potential in B cell malignancies (14–17). At present, research on CAR T cell for the treatment of AML has drawn considerable attention worldwide. Preclinical data have revealed several targets, such as CD44v6, FRβ, CD38, FLT-3, CD7, and CLEC12A. Targets, such as Lewis Y, CD33, CD123, and NKG2D-ligands, have been applied to clinical trials (18). To date, several groups have reported different clinical results of CD123-targeted CAR T cell (CART123). The first patient who received CART123 achieved a partial remission (PR) (19). Researchers from Cellectis recently reported two patients treated with UCART123, and both patients rapidly developed severe cytokine release syndrome (CRS) and capillary leak syndrome (CLS), one of whom died (20). The clinical results of “biodegradable” T cells that were electroporated with anti-CD123 CAR mRNA revealed no anti-tumor effect and toxicities other than fever or CRS (21). Although the outcome of Budde L's study exhibited that the hematopoietic toxicity of CART123 is quite limited, the CR rate is still needed to be improved (22). From the preliminary clinical results of CART123, the efficacy is much lower than that of CD19-targeted CAR T cell (CART19), possibly due to the specificity of targets. Therefore, the efficacy of CART123 remains to be improved. Considering the poor prognosis of *FUS-ERG*+ AML patients, a stronger treatment should be given.

Although a high objective response rate was achieved after CART19, the high relapse rate remains the major problem (23–25). It is gratifying that remission induced by CAR T cell can be consolidated by allo-HSCT (26–29). Therefore, CAR T cell is more likely to be a mean of bridging transplantation to enhance the efficacy of transplantation by eliminating tumors. Moreover, it can't be ignored that preclinical studies have demonstrated that CART123 causes severe cytopenia, and allo-HSCT could rescue the hematopoietic toxicity caused by CART123 in a mouse model (30, 31). Taken together, donor-derived CART123 was selected as part of conditioning regimen for haplo-HSCT to treat a patient with *FUS-ERG*+ AML relapse after allo-HSCT.

## Case Presentation

### Background of Patient

**Figure 1:**
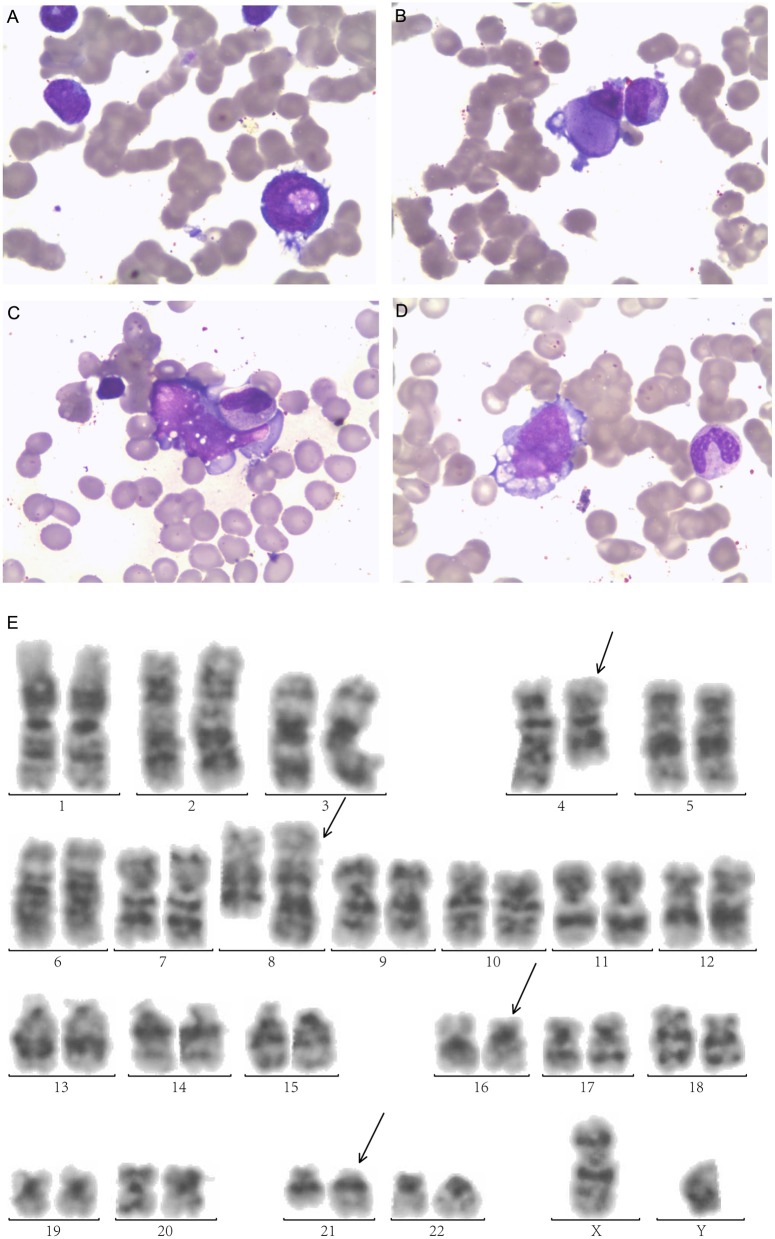
Morphologic examination of leukemic cells obtained from bone marrow aspirates exhibit **(A)** Leukemic cells with nucleonic vacuolation. **(B)** Megakaryocyte dysplasia **(C)** hemophagocytosis **(D)** cytoplasmic vacuolation. **(E)** G-banded bone marrow karyogram showing 46,XY,t(4;8)(q28;q24.1), t(16;21)(p11.2;q22). Black arrows indicate abnormal karyotypes.

A 25-year-old male was diagnosed with AML-M2 1 year ago, according to the French-American-British classification. Bone marrow (BM) morphology revealed 62.2% blasts, and peripheral blood (PB) was manually sorted by 39% blasts. The morphology examination exhibited megakaryocyte dysplasia, erythrophagocytosis, vacuolation in both cytoplasm and nucleus in leukemia cells in BM (Figures 1A–D).

Immunophenotyping by flow cytometry (FCM) analysis revealed positive results for CD34, CD38, HLA-DR, CD13, CD33, CD15, CD64, CD11b, CD56, CD117, CD123, MPO, and CyCD3 in BM. His karyotype showed 46, XX, t(4;8) (q28;q24.1,t(16;21) (p11.2;q22) (20) /46, XY (1) (Figure 1E). The FUS-ERG fusion gene was positive at 21.96% quantitatively in BM. He received induction chemotherapy DA (daunorubicin, cytarabine) and reinduction MA (mitoxantrone, cytarabine) and achieved CR. Then, he received two cycles of chemotherapy MA and IDA (idarubicin, cytarabine) and achieved minimal residual disease (MRD) negative by FCM. He received a human leukocyte antigen (HLA)-matched unrelated donor allo-HSCT after cyclophosphamide and total body irradiation (TBI) as preconditioning followed by Cyclosporine A (CsA), mycophenolate mofetil (MMF), basiliximab and short-term Methotrexate (MTX) for prophylaxis of graft-vs-host disease (GVHD). He achieved MRD-negative CR 1 month after HSCT but relapsed 2 months later.

**Figure 2:**
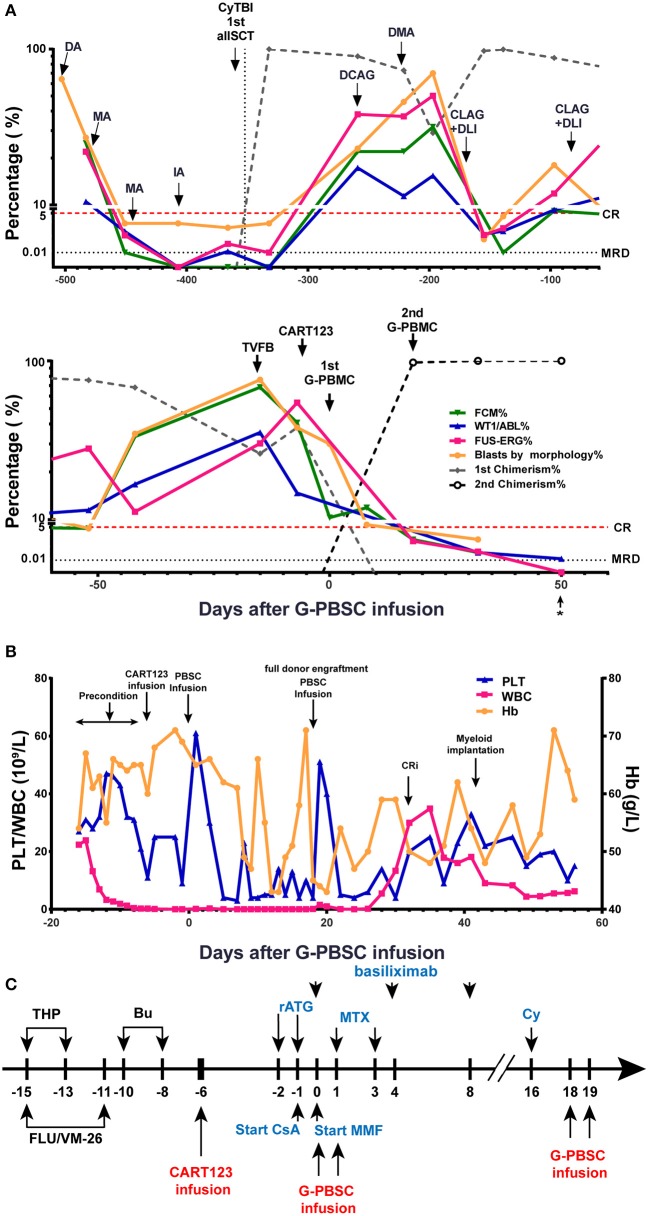
Evaluation of clinical response and hemogram changes during treatment. **(A)** Changes in tumor burden and donor chimerism during treatment in BM. * data from day 56 were obtained from PB samples because the BM samples were unobtainable. **(B)** Hemogram changes during treatment. **(C)** Process of donor-derived CART123 infusion and haplo-HSCT. The patient received a RIC regimen of TVFB and CART123 infusion. Given that the patient possibly developed an intense reaction during ATG infusion, basiliximab was administered. CsA/MTX/MMF was administered for GVHD prophylaxis after PBSC. Thus, PBSC was infused on day 0 and 1. Due to poor implantation, the patient received infusions of PBSC again on days 18 and 19. CART123, CD123-targeted chimeric antigen receptor (CAR) T cell; BM, bone marrow; FCM, flow cytometry; PB, peripheral blood; CRi, complete remission (CR) with incomplete blood count recovery; PLT, platelet; Hb, hemoglobin. DA, daunorubicin, cytarabine; MA, mitoxantrone, cytarabine; IDA, idarubicin, cytarabine; allo-HSCT, allogeneic hematopoietic stem cell transplantation; DCAG, decitabine, cytarabine, aclacinomycin, G-CSF; DMA, decitabine, mitoxantrone, Ara-c; CLAG, cladribine, Ara-c, G-CSF; DLI, donor lymphocyte infusion; TVFB, therarubicin, teniposide, fludarabine, busulfan; RIC, reduced-intensity conditioning; THP, therarubicin; FLU, fludarabine; VM-26, teniposide; Bu, busulfan; CsA, Cyclosporine A; MMF, mycophenolate mofetil; MTX, Methotrexate; Cy, Cyclophosphamide; haplo-HSCT, haploidentical hematopoietic stem cell transplantation; PBSC, peripheral blood stem cell; ATG, anti-thymocyte globulin; GVHD, graft-vs-host disease.

Then, he successively received DCAG (decitabine, cytarabine, aclacinomycin, G-CSF), DMA (decitabine, mitoxantrone, Ara-c), and CLAG (cladribine, Ara-c, G-CSF) combined with donor lymphocyte infusion (DLI) and achieved transient CR with MRD positive. He developed an anal fissure and perianal abscess, and the infection was controlled by anti-infective therapy. He subsequently relapsed 1 month later with central nervous system leukemia (CNSL) and was administered four cycles of Ara-c, MTX, and DXM by intrathecal injection and CLAG + DLI. CNSL was controlled, but the disease progressed (Figure 2A).

### Treatment of a Patient With CART123 as Part of Conditioning for Haplo-HSCT

**Figure 3:**
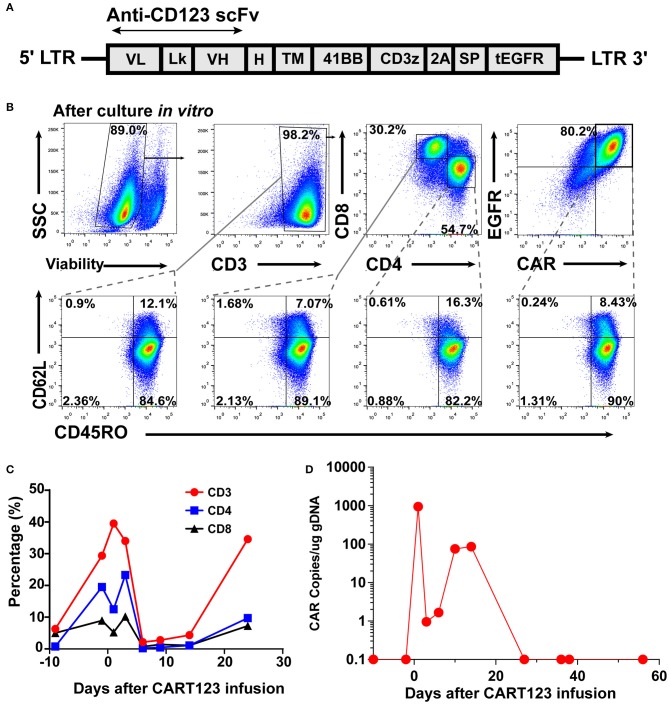
CAR design and expression; the expansion of CART123. **(A)** The structure of retroviral vectors encoding anti-CD123 CARs (32). The 41BB CAR consisted of anti-CD123 scFv FMC32716, a CD8a hinge region, CD8 transmembrane and cytoplasmic regions, and a CD3ζ cytoplasmic region. tEGFR was connected with the anti-CD123 CAR via the P2A peptide in this investigation. **(B)** CAR expression and CD3, CD4, CD8, CD45RO, and CD62L on T cells was detected by flow cytometry. CAR expression on T cells was detected by anti-EGFR antibodies. **(C)** Changes in the proportion of T lymphocytes in PB after CART123 infusion. **(D)** Changes in CAR copies in PB after CART123 infusion by qPCR. CAR, chimeric antigen receptor; CART123, CD123-targeted CAR T cell; PB, peripheral blood; qPCR, quantitative polymerase chain reaction; EGFR, human Epidermal Growth Factor Receptor.

The patient received reduced-intensity conditioning (RIC) regimen of TVFB (therarubicin 60 mg on d1 and 40 mg on d2-3; teniposide 200 mg on d1 and 150 mg on d3 and d5; fludarabine 50 mg on d1-5; and busulfan 3 mg/kg on d6-8) and CART123 1 day after preconditioning. The total infused CART123 was 1.1 × 10^8^ cells, and 9 × 10^7^ cells (CAR^+^ 80.2%) were CAR^+^ cells (1 × 10^6^/kg). This second-generation CAR consisted of anti-CD123 single chain fragment variable (scFv), CD8a hinge region, CD8 transmembrane domain, 41BB costimulatory domain, and CD3ζ cytoplasmic region. Truncated human Epidermal Growth Factor Receptor (EGFR) polypeptide (tEGFR) was integrated with CAR gene through a P2A peptide (Figure 3A). The viability was 89.0%, and the CD4^+^/CD8^+^ ratio was 1.81. Of the infused cells, 98.2% were CD3^+^ cells principally composed of the CD8^+^ subset (30.2%) and CD4^+^ subset (54.7%), and 8.43% and 90% of CAR+ cells were characterized with the central memory phenotype (CD45RO^+^/CD62L^+^) and effect memory phenotype (CD45RO^+^/CD62L^−^), respectively (Figure 3B). Both the stem cells and the CAR T cells were from his father, who exhibited a 5/10 HLA loci matching and ABO incompatibility with the patient. Subsequently, 4 days after CART123 infusion, anti-thymocyte globulin (ATG; 2.5 mg/kg/d 3d) was administrated for prophylaxis GVHD. However, during the second infusion of ATG, the patient developed tachypnea, tachycardia, and persistent hypoxemia. Given these serious side effects, the third-day infusion of ATG was canceled. Instead, the prophylactic regimen is adjusted to basiliximab (20 mg/d; days 0, 4, and 8), CsA, MTX (0.33g d1 0.02g d3, 6), mycophenolate mofetil (MMF; 1.8g 1/day 1.5g 1/night), and ATG (2.5 mg/kg/d 2d). Granulocyte colony-stimulating factor–mobilized peripheral blood stem cells (G-PBSC) was infused (mononuclear cells 11.77 × 10^8^/Kg, CD34^+^ 4.8 × 10^6^/Kg, CD3^+^ 4.1 × 10^8^/Kg) 6 days after CART123 infusion. Considering the potential hematopoietic toxicity of CART123, the second infusion of PBMC will be performed to promote implantation if the hematopoietic system remains unrecovered within about 14 days (Figure 2C).

### Response to Treatment

The blasts in BM decreased from 40.8 to 10.3% by FCM 6 days after CART123 infusion and decreased from 38 to 8% by morphology 14 days after CART123 infusion. On day 18, the second donor engraftment achieved 97.7% in BM. Although G-CSF was administrated to promote implantation from day 6, the hematopoietic system remains unrecovered until day 16. Thus, cyclophosphamide (4150mg) was administered as conditioning regimen and G-PBSC was infused again on day 18 and day 19 (mononuclear cells 14.28 × 10^8^/Kg, CD34^+^ 4.74 × 10^6^/Kg, CD3^+^ 4.44 × 10^8^/Kg). On day 32, blasts in BM were 0.5, 0.05, 0.042, and 0.02% by morphology, FCM, Wilms tumor-1 (WT1) and *FUS-ERG* detection, respectively. Compared to the first allo-HSCT, the second allo-HSCT was conducted in non-remission status using a RIC regimen, indicating the anti-leukemic activity of CART123. The patient achieved myeloid implantation on day 42 but was not weaned from platelet (PLT) and red blood cell (RBC) transfusion (Figures 2A,B).

### Expansion of CAR T Cell

The proportion of T lymphocytes (CD3^+^, CD4^+^, and CD8^+^) in PBMC was significantly increased after CART123 infusion. Then, T cells were sharply reduced after the administration of medications, such as methylprednisolone, ATG, and basiliximab (Figure 3C). Direct evidence of CART123 amplification was detected by qPCR (Figure 3D).

### Toxicities and Side Effects

#### CRS

**Figure 4:**
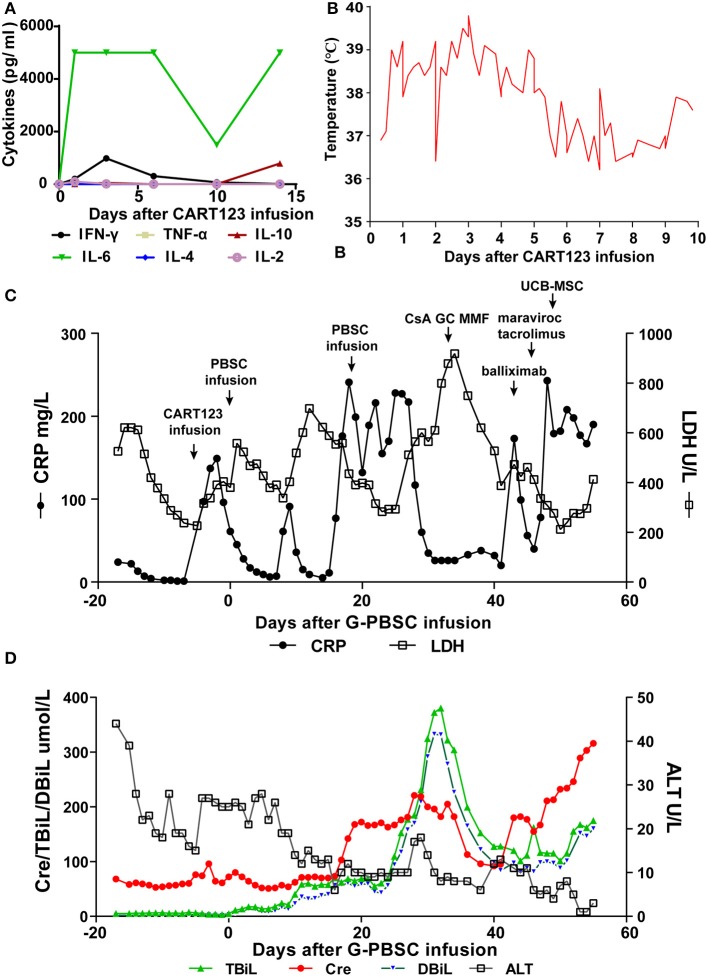
Trends of serum cytokines, body temperature, and major blood biochemical indexes after CART123 infusion. **(A)** Cytokines changed after CART123 infusion. Serum cytokine levels were measured at the indicated time points before or after CART123 and PBSC infusions. **(B)** Changes in body temperature after CART123 infusion. **(C)** Changes in CRP and LDH levels after G-PBSC infusion. **(D)** Changes in Cre, TBiL, DBiL, and ALT levels after G-PBSC infusion. CART123, CD123-targeted chimeric antigen receptor (CAR) T cell; PBMC, peripheral blood mononuclear cell; IL, interleukin; IFN, interferon; TNF, tumor necrosis factor; CRP, C-reactive protein; ALT, Alanine transaminase; aGVHD, acute graft-vs-host disease; CsA, Cyclosporine A; MMF, mycophenolate mofetil; GC, glucocorticoids; UCB-MSC, umbilical cord blood mesenchymal stem cells; DIC, disseminated intravascular coagulation; LDH, lactate dehydrogenase; Cre, creatinine; TBiL, total bilirubin; DBiL, direct bilirubin.

The patient developed a fever (>39°C), hypotension (92/58 mmHg) and pneumonia within 24 h after infusion, and these effects were evaluated as grade 3 CRS. He was immediately administered tocilizumab, a pressor agent and empirical anti-infective therapy. Assessment of cytokines in serum revealed an increasing trend for IL-6 and IFN-γ, and the effects in IL-6 was most obvious. Four days later, dyspnea, progressive pneumonia, and fever persisted (up to 41°C), and these features were evaluated as grade 4 CRS. The changing trend of C-reactive protein (CRP), lactate dehydrogenase (LDH), and body temperature was consistent with the level of cytokines and the clinical symptoms of the patient. Considering that tocilizumab on days −5 (240 mg) and −3 (400 mg) was invalid, methylprednisolone was administered from days −2 to 8 (day 4–7: 2 mg/kg for the first dose, 1 mg/kg q12h; d8-10: 2 mg/kg q12h) and the dose was gradually decreased. CRS was rapidly controlled after the infusion of methylprednisolone and ATG, with the decline of CRP, LDH, body temperature, and IFN-γ (Figures 4A–C).

#### Infections

The patient has an anal fissure before transplantation, and then it progressed to anal fistula with perianal infection after transplantation. However, the perianal infection caused repeated sepsis and pneumonia. Intermittent fevers occurred and were accompanied by sharp elevations in CRP and LDH after allo-HSCT. Repeated anti-infective, symptomatic and supportive treatment was administered to the patient and exhibited effective results. On day 28, he developed disseminated intravascular coagulation (DIC) due to infection and was controlled by the symptomatic treatment (Figure 4C).

#### GVHD

On day 32, after CRi was achieved, he soon developed fever, vomit, stomachache, and severe diarrhea. Total bilirubin (TBiL) progressively increased, mainly direct bilirubin (DBiL). He was diagnosed with aGVHD and administered by CsA, glucocorticoids (GC), MMF, basiliximab, tacrolimus, and maraviroc were successively for the treatment of aGVHD. On day 48, a total number of 7 × 10^7^ umbilical cord blood mesenchymal stem cells (UCB-MSC) were administered for the treatment of aGVHD. Finally, he was diagnosed with grade IV aGVHD involving liver and gut. In the final stage, creatinine increased progressively, reflecting the deterioration of renal function. Unfortunately, the patient died of aGVHD, severe pneumonia, intestinal obstruction, and multiple organ failure on day 56 (Figure 4D).

## Discussion

Still, relapse after allo-HSCT remained a ticklish question (33). In addition, for the FUS-ERG+ AML patients, who poorly response to standard treatment and have a dismal outcome, a novel therapy is urgently required (3). CART123 is promising immunotherapy targeting AML blasts and LSC. Compared to CART19, the preliminary clinical results of CART123 for patients with AML remained to be improved, possibly due to the specificity of targets. CART123 serves as a novel conditioning regimen to induce remission and bridges to transplantation is promising (34). However, the low remission rate limits this scheme. Also, prolonging the interval for transplantation may result in serious complications, such as infection and hemorrhage possibly caused by CART123 (30, 31). Thus, we designed and conducted a treatment using chemotherapy combined with donor-derived CART123 as a precondition of haplo-HSCT in a patient with FUS-ERG+ AML relapse after allo-HSCT. Theoretically, CART123 could kill the CD123+ LSC, which is resistance to chemotherapy, improving the effect of allo-HSCT. Meanwhile, immunosuppressors, including ATG and basiliximab, might kill or inhibit the proliferation of the CAR T cells (35, 36), and subsequent allo-HSCT could serve as a rescue for possibly hematopoietic toxicity of CART123.

Numerous reports suggested that second allo-HSCT may produce a better prognosis for patients with favorable performance status, remission at the time of the second transplant and most importantly, a long interval between initial transplant and relapse (37, 38). However, this *FUS-ERG*+ patient with AML relapsed within 3 months after the first allo-HSCT and maintained non-remission before the second allo-HSCT. The patient had progressive disease after a total of nine prior lines of therapy including ineffective DLI. Overall, this patient had an extremely dismal prognosis. He received a donor-derived CART123 as part of conditioning regimen for haplo-HSCT and achieved CRi status, full donor chimerism, and myeloid implantation. This is the first report of allogeneic CART123 as part of conditioning regimen for haploidentical HSCT in the treatment of relapsed AML after transplantation. This regimen could provide possible options for AML patients with an abysmal prognosis, including who is FUS-ERG positive.

CRS is the most common adverse effect after CAR T cell treatment, with an incidence ranging from 18 to 100% while severe CRS from 8 to 46% in the previous major CART19 clinical studies (39). Onset of CRS is approximately 1-6 days after CAR T cells infusion, with >95% of CRS occurring before day 12 following CAR T infusion (40). After CART123 infusion, this patient rapidly developed grade 4 CRS and controlled by glucocorticoid and other immunosuppressors. Although CRS was mostly controlled before PBMC infusion, a high level of IL-6 still exists. It can be seen from the previous study that the decrease in IL-6 levels lags behind other cytokines (41). It is reported that the development of CRS is related to factors significantly impacting *in vivo* CAR T cell expansion, such as disease burden, intensity of precondition regimen, the CAR T cell dose, and design of CAR (40, 42, 43). Thus, the rapidly occurred severe CRS of our patient was possibly related to high disease burden and intensive chemotherapy. It still remains unknown whether the antigen target of the CAR affects the rate of CRS (43). However, CD123 is the α subunit of IL-3 receptor, which might have critical roles in inflammation and anti-apoptosis (44–48). As previously reported, severe CRS and CLS also occurred in two patients in Cellectis' UCART123 clinical trials. Our previous study demonstrated that the expression of CD123 on endothelial cells could be upregulated when co-cultured with CART123 and IFN-γ/TNF-α could aggravate endothelial damage caused by CART123 *in vitro*, although the underlying mechanism needs further study (32). Therefore, the upregulation of CD123 expression under inflammation or apoptosis possibly aggregate CRS and even CLS, which need to pay more attention in subsequent research. Tocilizumab is now the FDA-approved standard treatment and widely used for CAR-T-cell-induced severe or life-threatening CRS, and a dose of 8 mg/kg is recommended for patients ≥30 kg, although the optimal dose is undefined (49, 50). Seventy percentage of patients responded to 1–2 doses of tocilizumab within 14 days, with a median time to response of 4 days (49, 50). Thus, additional immunosuppression with corticosteroids is needed in some cases of severe CRS refractory to tocilizumab. Although corticosteroids suppress T-cell function and/or induce T-cell apoptosis (51–53), it does not affect short-term anti-tumor efficacy of CART19 (54, 55). Recently, Gardner et al. (56) shown that early intervention with the use of tocilizumab and/or corticosteroids in subjects with early signs of CRS reduces the frequency of CRS without attenuation anti-leukemic potency of CART19. Therefore, early, adequate, full-course treatment for CRS may benefit patients more.

However, the patient developed several complications after allo-HSCT, including infections, poor graft function (PGF), and GVHD, which closely related to his disease status. Park et al. (57) showed that the presence of grade ≥3 CRS was a factor independently associated with any infection especially bloodstream infection, but whether tocilizumab or corticosteroids used to treat high-grade CRS increases the risk of infection independent of CRS remain unknown. Also, Hill et al. (58) demonstrated most infections occurred early after CAR T cells infusion and CRS severity was the only factor after CAR T cells infusion associated with infection in a multivariable analysis. Tocilizumab has been shown to confer increased risk of cytopenias and infections in patients with rheumatoid arthritis (59). Until now, although both corticosteroids and/or tocilizumab may increase infection risk in patients with severe CRS (58), further study will be required.

PGF, which can be a life-threatening complication, occurs in 5–27% of patients after allo-HSCT (60–63). The patient experienced a primary PGF after allo-HSCT. He achieved full donor chimerism and myeloid implantation while presented with thrombocytopenia and erythropenia associated with a hypercellular marrow after transplantation. The occurrence of PGF in this patient after allo-HSCT may be related to major ABO incompatibility, HLA mismatching, GVHD, the RIC regimen, and septicemia, according to previous studies (61, 63–66). Moreover, CART123 remained detectable 14 days after CART123 infusion. Although the persistence of CART123 is short, it is consistent with the reduction in tumor burden and the occurrence of CRS. It may be due to the use of ATG and basiliximab that might kill or inhibit the proliferation of the CAR T cells (35, 36). This result indicated that CAR T-cell ablation using cetuximab, an anti-EGFR antibody (67), is not necessary before transplantation. Although full donor chimerism was achieved in the presence of CART123, the myeloid implantation was achieved after the second infusion of PBMC, accompany with the CAR copies undetectable. Thus, the persistence of CART123 may affect the development and differentiation of hematopoietic cells. Therefore, the interval between the infusion of CART123 and G-PBSC need to be explored in further research, to guarantee the elimination of CART123 before allo-HSCT.

In this study, the patient developed a fatal aGVHD after achieved CRi. It is most likely related to recurrent infections after transplantation. Infectious diseases can theoretically promote the elevation of inflammatory cytokines after allogeneic HSCT and the activation of various immune effector cells, which might lead to aggravation of acute GVHD (68). Unfortunately, perianal infections in this patient were challenging to be effectively eliminated and caused repeated sepsis and pneumonia, possibly leading to severe aGVHD, and eventually death due to multiple organ failure. Moreover, higher CD3+ doses had an increased incidence of aGVHD or grade III-IV aGVHD in allo-HSCT (69, 70). The reinfusion of G-PBSC to promote engraftment may aggravate the occurrence of aGVHD in this patient. Hence, many factors, such as infections, CD3+ cell doses, and disease status, possibly results in the occurrence of aGVHD. Anwer et al. (71) found that GVHD occurred in only 6.9% of patients who relapsed after HSCT and received donor-derived CART19. It can be seen that the incidence of GVHD after donor-derived CART19 is lower in patients who have relapsed after transplantation. However, it remains to be seen whether the infusion of a haploidentical CAR T cell to a patient who has not been transplanted will produce GVHD. Haploidentical CAR T cell has been used as part of a pretreatment regimen to treat B-ALL and achieved full donor engraftment, with a mild “GVHD-like” reaction or no GVHD (35, 72). A haploidentical CAR T cell without previous allo-HSCT had a clinically significant antitumor activity without serious side effects (73). Therefore, according to these preliminary studies, haploidentical CAR T cell for B cell malignancies has a low risk of GVHD. In the current study, CAR T cell was undetectable by q-PCR when GVHD occurred. However, unlike CD19, which is restricted to B cells, CD123 is customarily expressed in kinds of hematopoietic and non-hematopoietic cells, especially endothelial cells (12, 13, 44). The expression level of CD123 on T and endothelial cells even could be upregulated under proliferation and cytokines induction, respectively (44, 74). Theoretically, CART123 might have a stronger off-target effect and is more prone to a wide range of inflammatory cytokines release compared to CART19, thereby aggravating GVHD. In conclusions, the influencing factors of GVHD in this patient are complicated, of which CART123 is not excluded. The CRS possible off-target effect and of CART123 and its effects on GVHD needs further study.

Taken together, our results hint that haploidentical CART123 reduces the chemotherapy-resistant AML blasts for *FUS-ERG*-positive AML without affecting the full donor chimerism and myeloid implantation. However, the long-term anti-leukemic effect, the interval between infusions of CART123 and G-PBSC, and the prophylaxis of GVHD still require further study.

## Materials and Methods

See the Materials and Methods section in the Supplementary Material.

## Data Availability Statement

All datasets generated for this study are available on request to the corresponding author.

## Ethics Statement

The studies involving human participants were reviewed and approved by Institutional Review Board at the Affiliated Hospital of Academy of Military Medical Sciences (Beijing, China). The patients/participants provided their written informed consent to participate in this study.

## Author Contributions

CH and ZB designed the trial and experiments and analyzed the data. HL and CJ performed experiments, analyzed the data, and revised the manuscript. SY performed experiments, analyzed the data, and wrote the paper. LY, LB, WQ, NH, FH, ZL, and WP performed experiments and analyzed the data.

### Conflict of Interest

LY, FH, ZL, and WP was employed by the company HRAIN Biotechnology. The remaining authors declare that the research was conducted in the absence of any commercial or financial relationships that could be construed as a potential conflict of interest.
