# Supplementary material for: Donor-Derived CD123-Targeted CAR T Cell Serves as a RIC Regimen for Haploidentical Transplantation in a Patient With FUS-ERG+ AML
Source: Front Oncol. 2019 Dec 3;9:1358. doi: 10.3389/fonc.2019.01358 (PMC6901822; doi:10.3389/fonc.2019.01358)
Supplement: Supplementary file 1 [file Data_Sheet_1.PDF]

Materials and Methods

Generation of retroviral vectors

The retroviral vectors encoding anti-CD123 CARs were constructed based on a modified Moloney Murine Leukemia Virus (Mo-MLV) vector described previously [1]. This second-generation CAR consisted of anti-CD123 single chain fragment variable (scFv), CD8a hinge region, CD8 transmembrane domain, 41BB costimulatory domain, and CD3ζ cytoplasmic region. Truncated human Epidermal Growth Factor Receptor (EGFR) polypeptide (tEGFR) was integrated with CAR gene through a P2A peptide. The production of clinical-grade retroviral was described previously [2].

CAR T cell production

Thawed PBMC from the eligible donor were cultured in T cell medium (TCM) containing X-vivo15 serum-free medium (Lonza, Allendale NJ), 5% (vol/vol) GemCell human serum antibody AB (Gemini Bio Products, West Sacramento CA), 1% (vol/vol) Glutamax-100× (Gibco Life Technologies), 10mM HEPES buffer (Corning), and 2% （m/V） N-Acetyl-L-cysteine (Sigma). The culture was supplemented with 50-100 IU/mL human IL-2. The PBMC were activated and expanded using Dynabeads human T-expander CD3/CD28 (Life technology) at a bead:T cells ratio of 1:1. The retroviral supernatants were spin-loaded onto non-tissue culture-treated 24-well plates coated with 15 μg/ml RetroNectin (Takara)per well by centrifuging 2h at 2000g at 32°C. Activated PBMC were resuspended at the concentration of 5×10^5cells/mL in TCM,

containing 50 IU/ml recombinant human IL-2, and then added to the vector-loaded 24-well plate. The plates were spun at 1000g at 32°C for 10 minutes and incubated at 37°C overnight. During ex vivo expansion, culture medium was replenished, and T cell density was maintained between 0.5 and 1 × 10^6 cells/mL. We added fresh medium and cytokines every other day. At days 8-12, the CAR T cell was ready for the production release testing: CAR+ >30% and expansion at least 5-fold under the stimulation of CD3/28 beads stimulation in vitro. Then, we could prepare for infusion or thawing at the appropriate time.

Biomarker analysis

Multi-parametric flow cytometry was used for analysis of various PBMC and CART123 samples. The CART123 were stained with fluorescent-labeled antibodies against CD3, CD4, CD8, CD45RO, and CD62L (BioLegend, San Diego, CA). tEGFR was detected with anti-EGFR antibodies (BioLegend, San Diego, CA) to directly represent the detection of CAR.

The presence, expansion, and persistence of CART123 in the blood were monitored by quantitative PCR. Genomic DNA was isolated from PBMC samples using MiniBEST Universal Genomic DNA Extraction Kit (Takara), quantified by a spectrophotometer, and stored at -80°C. The qPCR analysis on genomic DNA samples was performed to detect the integrated CD123CAR transgene sequence using the following primer pair and a specific probe.

CD123CAR Forward primer: 5’-ACCATCAACCCCGTGGAA-3’

CD123CAR Reverse primer: 5’-TCGTTGCTCTGCTGGCAGTA-3’

CD123CAR probe: 5’-FAM-CCGACGACGTGGCC-3’

A parallel qPCR detection on the CDKN1A gene (Genebank: Z85996) was performed as previously described [2].

Cytokine levels of IL-2, IL-4, IL-6, IL-10, TNF-α, and IFN-γ were measured from serum samples collected by flow cytometry using the Cytometric Bead Array (BD Biosciences), according to manufacturer's instructions.

[1] B. Engels, H. Cam, T. Schüler, S. Indraccolo, M. Gladow, C. Baum, T. Blankenstein, and W. Uckert, Retroviral vectors for high-level transgene expression in T lymphocytes. Human Gene Therapy 14 (2003) 1155.

[2] Z. Cheng, R. Wei, Q. Ma, L. Shi, F. He, Z. Shi, T. Jin, R. Xie, B. Wei, J. Chen, H. Fang, X. Han, J.A. Rohrs, P. Bryson, Y. Liu, Q.J. Li, B. Zhu, and P. Wang, In Vivo Expansion and Antitumor Activity of Coinfused CD28- and 4-1BB-Engineered CAR-T Cells in Patients with B Cell Leukemia. Mol Ther (2018).
